# Protocol for the BAG-RECALL clinical trial: a prospective, multi-center, randomized, controlled trial to determine whether a bispectral index-guided protocol is superior to an anesthesia gas-guided protocol in reducing intraoperative awareness with explicit recall in high risk surgical patients

**DOI:** 10.1186/1471-2253-9-8

**Published:** 2009-11-30

**Authors:** Michael S Avidan, Ben J Palanca, David Glick, Eric Jacobsohn, Alex Villafranca, Michael O'Connor, George A Mashour

**Affiliations:** 1 Department of Anesthesiology, Washington University, St Louis, USA; 2 Department of Anesthesia & Critical Care, University of Chicago, Chicago, USA; 3 Department of Anesthesia, University of Manitoba, Manitoba, Canada; 4 Division of Neuroanesthesiology, University of Michigan, Michigan, USA

## Abstract

**Background:**

Awareness with explicit recall of intra-operative events is a rare and distressing complication that may lead to severe psychological symptoms. Candidate depth of anesthesia monitors have been developed, partly with the aim of preventing this complication. Despite conflicting results from clinical trials and the lack of incisive validation, such monitors have enjoyed widespread clinical adoption, in particular the bispectral index. The American Society of Anesthesiologists has called for adequately powered and rigorously designed clinical trials to determine whether the use of such monitors decreases the incidence of awareness in various settings. The aim of this study is to determine with increased precision whether incorporating the bispectral index into a structured general anesthesia protocol decreases the incidence of awareness with explicit recall among a subset of surgical patients at increased risk for awareness and scheduled to receive an inhalation gas-based general anesthetic.

**Methods/Design:**

BAG-RECALL is a multi-center, randomized, controlled clinical trial, in which 6,000 patients are being assigned to bispectral index-guided anesthesia (target range, 40 to 60) or end-tidal anesthetic gas-guided anesthesia (target range, 0.7 to 1.3 age-adjusted minimum alveolar concentration). Postoperatively, patients are being assessed for explicit recall at two intervals (0 to 72 hours, and 30 days after extubation). The primary outcome of the trial is awareness with explicit recall. Secondary outcomes include postoperative mortality, psychological symptoms, intensive care and hospital length of stay, average anesthetic gas administration, postoperative pain and nausea and vomiting, duration of stay in the recovery area, intra-operative dreaming, and postoperative delirium.

**Discussion:**

This trial has been designed to complement two other clinical trials: B-Unaware and MACS (ClinicalTrials.gov numbers, NCT00281489 and NCT00689091). With the large patient numbers and complementary rigorous designs, it is envisaged that pre-specified meta-analyses will address some of the outstanding controversies and questions relating to processed electroencephalography monitoring.

**Trial registration:**

ClinicalTrials.gov Identifier: NCT00682825

## Background

Many patients facing surgery dread the prospect of being awake, in pain and unable to move owing to inadequate general anesthesia. Awareness during anesthesia with subsequent explicit recall (AWR) is distressing and may contribute to posttraumatic stress disorder [1-4]. Several monitors, mostly based on processed electroencephalograph (EEG) information or auditory evoked potentials, have been developed in an attempt to measure depth of anesthesia or depth of hypnosis[5]. It is hoped that the use of such monitors during general anesthesia will decrease the likelihood of AWR. Of these monitors, the bispectral index (BIS monitor) has been most widely adopted in clinical practice. The BIS monitor incorporates a proprietary algorithm based on signals from a processed scalp EEG. The monitor shows a dimensionless number between 0 and 100, with lower numbers reflecting deeper anesthesia[6,7].

The American Society of Anesthesiologists (ASA) has issued a practice advisory on AWR[8]. The ASA advisory does not currently advocate the routine use of brain monitors such as the BIS monitor to prevent AWR as the evidence remains equivocal[8]. According to a Joint Commission for Accreditation of Healthcare Organization (JCAHO) Sentinel Event Alert, between 20,000 and 40,000 cases of anesthesia awareness may occur each year in the USA alone[9]. The JCAHO Alert states that the use of BIS monitoring to help guide anesthetic administration may be associated with a reduction in the incidence of AWR in adults during general anesthesia and sedation[9].

A large multi-center observational study in the USA showed that, despite modern anesthesia techniques, the overall incidence of AWR in the general surgical population remains about 0.1-0.2%[4]. In this study, the incidence of AWR was not lower when a BIS monitor was used, but there was no protocol for anesthesia based on the BIS monitor and anesthesia care was not standardized among centers or practitioners. Some patients are at higher risk for AWR according to the type of surgery, co-morbidities, anesthetic techniques (including total intravenous anesthesia (TIVA)), chronic medications, substance misuse, and unidentified genetic factors[8,10-13]. For some of these patients the risk of AWR has been estimated to be as high as 1%[10].

The 2,500 patient multi-center B-Aware Study was designed to determine whether the BIS monitor might substantially decrease the incidence of AWR among patients at higher risk for AWR[10]. The study was powered to detect a large 0.9% absolute reduction in AWR in this population because the introduction of BIS monitoring into routine clinical practice would require proof of clinical efficacy[10,14]. The B-Aware Study found that incorporation of a BIS-guided protocol decreased the incidence of 'definite' AWR among patients who were at higher risk by 0.74% (95% CI, 0.08% to 1.5%)[10]. Unlike the multi-center study in the USA referred to above [4], in the B-Aware Study anesthesiologists followed a structured protocol when the BIS was used, striving to titrate anesthesia such that the BIS number was between 40 and 60 for most of the anesthetic period[10]. An alternative hypothesis explaining the results of the B-Aware Study is that the directive to record BIS values at pre-specified intervals might have heightened the overall level of vigilance about awareness in the intervention group. Care was not protocol-driven in the control group, and usual practice was followed. Another potential confounder in the B-Aware Study was the pooling of patients who received TIVA with those who received volatile anesthesia, since the incidence of AWR and the efficacy of BIS monitors may differ between the two techniques[11,12].

An attempt was made to address some of the potential confounders in the B-Aware Study in the prospective randomized B-Unaware trial, which enrolled 2,000 patients at higher risk for AWR, all of whom received inhalation anesthesia[15,16]. Protocols to increase vigilance during anesthetic delivery were based on either BIS monitoring or on end-tidal anesthetic concentration. In the BIS monitored group, volatile anesthetics were titrated to target the manufacturer's recommended BIS range for surgical anesthesia. In the control group, the end-tidal anesthetic agent target was above 0.7 minimum alveolar concentration (MAC), a concentration that may suppress the formation of emotionally charged memories[16]. Audible alarms alerted practitioners when targets were not achieved. There was no difference in the incidence of 'definite' AWR between the groups, with two cases occurring in each (difference = 0%; 95% CI, -0.56 to 0.57%)[15]. The results of the B-Unaware trial suggest that for patients at higher risk for awareness undergoing inhalation anesthesia with a protocol designed to increase vigilance, the BIS monitor is very unlikely to decrease the incidence of 'definite' AWR by 0.9%. But the precision or confidence interval does not exclude the possibility that BIS may still be associated with a clinically important reduction in AWR in this setting of up to 0.57%.

There were additional findings arising from the B-Unaware Trial, including the following: 1) The BIS protocol was not associated with decreased administration of anesthesia; 2) Cumulative duration of BIS <45 was independently associated with increased mortality, independent of anesthetic dose and protocol arm; 3) The BIS protocol was not associated with decreased long term psychological symptoms; 4) The BIS protocol was not associated with more rapid postoperative recovery or decreased side effects of anesthesia; 5) Dreaming was not associated with AWR, with mean anesthetic dose, with mean BIS values, or with the use of the BIS monitor; 6) The BIS protocol was not associated with decreased intensive care stay, decreased hospital stay or decreased mortality. These findings should be regarded as hypothesis generating and requiring verification or refutation in subsequent trials.

It is important for patients, for regulatory bodies and for anesthesia practitioners that brain monitors such as the BIS undergo rigorous evaluation to determine whether they really do decrease AWR and whether they are cost effective before they are adopted into routine practice. The primary purpose of the BAG RECALL trial will therefore be to answer this question with greater precision in patients at high risk for AWR undergoing general anesthesia with a potent inhalation agent. Our companion study, the Michigan Awareness Control Study (MACS, NCT00689091), is designed to answer this question rigorously in the general surgical population, regardless of risk for AWR. The secondary outcomes of BAG-RECALL will address with increased accuracy the additional findings of the B-Unaware Trial, and other important questions. We also plan meta-analyses including the B-Unaware, MACS and BAG-RECALL trials.

## Methods/Design

### Patients

This is a multi-center study that has been approved by the Human Studies Committees at Washington University in St Louis, University of Chicago, and University of Manitoba. The Consolidated Standards of Reporting Trials guidelines will be followed in the conduct of the study and in the reporting of results[17,18]. We are evaluating patients, who are older than 18 years and who are undergoing surgery, for eligibility prior to their surgery on the basis of preoperative assessment records. Patients are required to be at high risk for AWR, and to have general anesthesia with isoflurane, sevoflurane, or desflurane, without supplemental nitrous oxide. The criteria for identifying patients at high risk for AWR have been based on previous studies, reviews, and guidelines. Patients at high risk are defined as those with at least one risk factor. The pre-specified risk factors are preoperative long-term use of anticonvulsant agents, opiates, benzodiazepines, or cocaine; a cardiac ejection fraction less than 40%; a history of AWR; a history of difficult intubation or anticipated difficult intubation; ASA physical status class 4 (those who have systemic disease that is a constant threat to life) or class 5 (those who are not expected to survive without the operation); aortic stenosis; end-stage lung disease; marginal exercise tolerance not resulting from musculoskeletal dysfunction; pulmonary hypertension; planned open-heart surgery; and daily alcohol consumption. Patients are excluded if the surgical procedure or positioning of the patient prevents BIS monitoring or if the surgery requires a wake-up test. Also excluded are patients who have dementia, who are unable to provide informed consent, or who have a history of stroke with residual neurological deficits.

### Study Design

**Figure 1:**
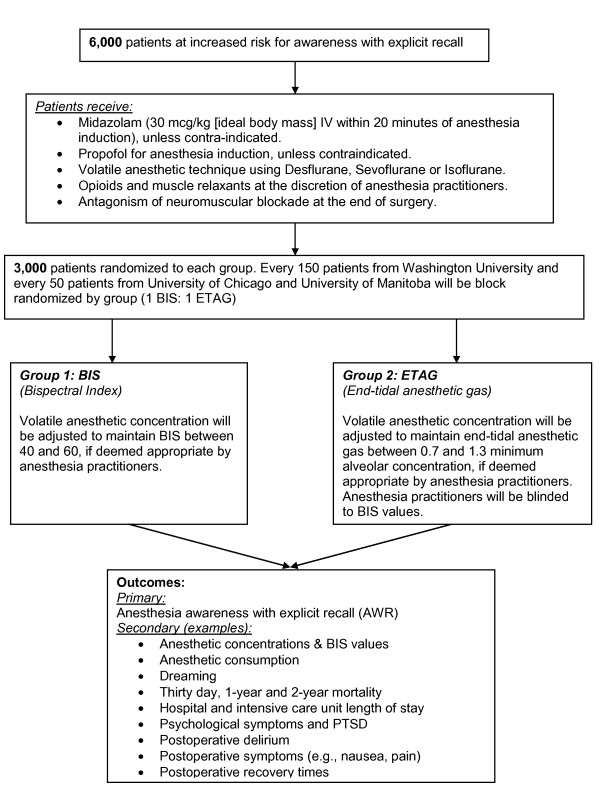
**Conduct of the Trial**.

The design is a multi-center, prospective study, in which 6,000 patients are undergoing pre-randomization electronically in blocks of 100, with 50 patients assigned to a BIS-guided protocol and 50 to an end tidal anesthetic gas (ETAG)-guided protocol. Enrollment of 4,500 patients is planned at Washington University, and enrollment of 750 patients each are planned for inclusion at University of Chicago and at University of Manitoba. (Figure 1) Eligible patients are undergoing randomization after providing written informed consent. The anesthesia practitioners are aware of the assignments of the patients, but the patients, the postoperative interviewers, the expert reviewers, and the statistician are not.

The manufacturer of the BIS monitor (Aspect Medical Systems) had no role in the study design, and will likewise not be involved in data collection, data analysis, or in manuscript preparation. No study monitors or other means of support are being provided by Aspect Medical Systems.

### Procedures

A BIS Quatro Sensor (Aspect Medical Systems) is applied to the left side of the forehead of each patient. The anesthesia practitioners caring for the patients in the ETAG group are using a monitor configuration that omits the BIS number, so they are unaware of the BIS values. The practitioners in both groups can view the ETAG concentrations. Laminated double-sided summaries of the protocols (Appendix 1) are being distributed to practitioners to provide education and to enhance compliance.

In the BIS group, an audible alarm is being set to indicate when the BIS values exceed 60 or fall below 40; no ETAG alarms are being set in the BIS group, and there is no recommendation to maintain the ETAG concentration within any range. In the ETAG group, an audible alarm is being set to indicate when the ETAG concentration falls below 0.7 age adjusted MAC or exceeds 1.3 age adjusted MAC[19,20]. In the event that alarm settings are unavailable for ETAG concentration, inspired anesthetic gas alarms are set. During cardiopulmonary bypass, the anesthetic-gas concentration is being measured from the effluent of the cardiopulmonary-bypass machine[21]. A sign is being affixed to the anesthesia machines reminding the practitioners to check the BIS value or ETAG concentration and to consider whether the patient might have intraoperative awareness. BIS values and ETAG concentrations are being collected at a minimum frequency of 1-minute intervals either by electronic anesthesia recording using Metavision^® ^software (iMDsoft, Needham, MA), or by direct electronic transfer to Microsoft Excel^® ^(Microsoft, Redmond, WA), or by use of proprietary TrendFace Solo^® ^software (ixellence GmbH, Wildau, Germany). Manual records of anesthesia and digital photographs of monitor trends are being used as alternatives in the rare instances that the computer data or the electronic anesthesia records are incomplete. A composite Microsoft Access^® ^(Microsoft, Redmond, WA) database is being assembled, which will incorporate all the data from the three sites and will facilitate subsequent analysis.

AWR is being assessed with the use of the modified Brice questionnaire with targeted supplementary questions (Appendix 2). The investigators are unaware of the patients' assignments and of previous assessments of their anesthesia awareness. Patients are contacted within 72 hours of their surgery and at 30 days after extubation. Previous studies have shown that AWR is not reliably detected with only a single, early postoperative interview[10,15]. A different investigator, who is also blinded as to whether patients were allocated to the BIS or ETAG protocol, contacts patients who report memories of the period between "going to sleep" and "waking up" at either interview, and further structured questions are asked (Appendix 3). Patients who report such memories are all offered referral for expert counseling.

After all patients have completed the study, three experts who are unaware of the assignments of the patients will independently review the questionnaire responses and classify each patient into one of three groups: definite AWR, possible AWR, and no AWR. In assessing whether AWR has occurred, the experts will be instructed to focus on memories of events that could occur only in the operating room during the anesthetic and surgical periods. The outcome will be determined when at least two of the experts are in agreement. If two experts hold opposing views, a fourth expert will be asked to review the questionnaires. The experts will assign each awareness event to one of the categories on the Michigan Awareness Classification Instrument, which has been shown to have excellent inter-rater agreement[22].

For each patient with either possible or definite AWR, an investigator blinded to the intervention group will use the accounts given by the patients and information in the anesthetic record to identify a time window during which anesthesia awareness was likely to have occurred. When a specific time window cannot be identified, the entire anesthetic period will be considered.

### Secondary Outcomes

A number of secondary outcomes, including several of the preliminary findings of the B-Unaware Trial, will be addressed by the BAG-RECALL Trial. Some of the secondary outcomes will also be addressed through meta-analysis including the B-Unaware, BAG-RECALL and MACS clinical trials. The secondary outcomes are listed on the clinical trials registration site for BAG-RECALL.

### Statistical Analysis

The primary outcome measure of the study will be the incidence of AWR in the BIS and ETAG groups. The null hypothesis of the BAG-RECALL trial is that the BIS-guided protocol will not decrease AWR compared with the ETAG-guided protocol in this population. The alternative hypothesis, for which the study is powered, is that the BIS monitor confers a clinically significant advantage in decreasing AWR, independent of the protocol-based care. We based our projected estimates of AWR incidence among higher risk patients on the results of the B-Aware and the B-Unaware trials. With an incidence of AWR of 0.5% (5 in a 1,000) in the ETAG group and 0.1% (1 in a 1,000) in the BIS group, 3,000 patients in each group would be sufficient to detect this difference with a one-tailed alpha of 0.05 using Fisher's exact test (power = 87%). This represents an absolute risk reduction of 0.4% and a number needed to treat of 250 patients at higher risk for AWR. Any lesser absolute risk reduction would probably be clinically unimportant and not cost effective with the present cost of disposable BIS electrodes. We will also use a one-sided Fisher's exact test to determine if the BIS-guided group has a lower incidence of definite and possible AWR. Confidence intervals for absolute risk reduction will be calculated with the use of Newcombe's method without continuity correction. There will be no interim analysis. Chi-Squared, Fisher's exact, unpaired Mann-Whitney, and unpaired t-tests will be used as appropriate for other comparisons between the groups, including patient characteristics and secondary outcomes. Intention-to-treat analysis is planned. A multivariable regression model will be used to evaluate the independent association of specific variables with AWR and secondary outcomes. Agreement among experts assessing AWR will be assessed using a two-way random effects intraclass correlation (ICC) coefficient for absolute agreement using the metric: no = 0, maybe = 1, yes = 2. Apart from assessment of AWR, all other significance testing will be two-sided and a P value less than 0.05 will be considered to indicate statistical significance.

## Discussion

This trial has been designed to complement two other clinical trials: B-Unaware[15] (NCT00281489) and MACS (NCT00689091). With the large patient numbers and complementary rigorous designs, it is envisaged that pre-specified meta-analyses will address some of the outstanding controversies and questions relating to the potential usefulness of processed EEG monitors in relation to preventing AWR and in relation to other clinically important outcomes.

Some important issues will remain unaddressed by BAG-RECALL, such as: 1) Are processed EEG monitors, such as BIS, useful in the setting of total intravenous anesthesia? 2) Is BIS the most useful processed EEG monitoring technique? 3) Do processed EEG monitors increase the likelihood of AWR if they are used specifically to minimize anesthetic dose? 4) What impact does the BIS protocol have in settings other than the one in which this trial is being conducted? 5) Are processed EEGs cost effective if used routinely for all patients requiring general anesthesia? 6) Does the use of nitrous oxide impact the likelihood of AWR? As BAG-RECALL is enrolling only patients who are considered to be at higher risk for AWR, and who are undergoing general anesthesia with a potent inhalation agent, the results may not be generalized beyond this patient population. If the BIS protocol is associated with a reduction in AWR, it does not rule out the possibility that it would be associated with no reduction or even an increased incidence of AWR in other patient populations. This emphasizes the importance of our companion trial, MACS (NCT00689091), which is enrolling all surgical patients, regardless of perceived risk.

An important assumption underlying the design of the BAG-RECALL trial is that risk factors for AWR are known and patients with increased risk can be identified and enrolled. However, if there are unidentified risk factors for AWR, such as genetic resistance to the hypnotic or amnestic actions of anesthetic agents, and if these are relatively common compared with the risk factors that have been used to identify eligible patients for BAG-RECALL, this could be an important hidden confounder. If the unidentified risk factors are distributed unequally between the BIS and ETAG groups, this could confound the results of the trial independent of the BIS and ETAG protocols. With rare risk factors and an even rarer outcome (i.e., AWR), it is not certain that randomization alone will result in equal assignment of patients with hidden risk factors to each group.

One of the most important reasons to prevent AWR is to prevent long-term psychological symptoms and the development of PTSD. While assessing psychological consequences is an important secondary outcome of BAG-RECALL, it may not be valid to apply the results of BAG-RECALL to patients who suffer AWR in general. The reason for this is that all patients who experience AWR are referred to an expert counselor whose intervention may mitigate the psychological sequelae of AWR.

A criticism leveled against the B-Unaware trial was that the control group also used a protocol, which was not reflective of current standard practice. For the B-Unaware, the BAG RECALL, and MACS clinical trials, we have designed protocols that are simple and easy to implement at no cost over current practice. We have stressed that the protocols are not intended to be restrictive, but are merely intended to increase vigilance and possibly to provide earlier warning when awareness is more likely to occur. If processed EEG monitors, such as the BIS, are to enjoy widespread adoption, they must confer advantage over the best, simple alternative practice, not just usual care.

## Competing interests

The authors have no financial or non-financial competing interests to declare. This study has no industry funding or support.

## Authors' contributions

MSA is the principal investigator of the study and was the primary author of the manuscript. BJP, DG, EJ, AV, MOC and GAM were all involved in study design and made contributions to the manuscript. All authors have read and approved the final version. All the members of the BAG-RECALL Study Group are participating actively in patient enrollment, follow-up interviews, data collection and data entry.

## Appendix 1: Protocols

### BIS Protocol

1) The protocol is intended as a **rough guide**, not to be prescriptive.

2) BIS **alarms **are set to alert, not to restrict.

3) The BIS is intended to guide practitioners in **two chief ways**:

a. Provide **sufficient anesthesia **for "hypnotic" component GA.

b. Decrease anesthesia **safely to minimize **the mean anesthetic dose.

4) Ideally anesthesia should be titrated such that the BIS is maintained between 40 and 60, or alternatively, between **45 and 60 **during surgery. During **skin closure**, anesthesia may be lightened, ideally keeping the **BIS < 75**.

5) The BIS should be **interpreted in context**, taking into account patient characteristics, surgical stimulation, hemodynamic parameters, analgesic administration, use of regional anesthesia, extent of muscle relaxation, anesthetic gas concentration, and other data provided by the BIS monitor.

6) Reliance on the BIS alone is not recommended. **Artifacts **and poor signal quality may lead to inappropriate BIS values. Artifacts may be caused by poor skin contact (high impedance), muscle activity or rigidity, head and body motion, sustained eye movements, improper sensor placement and electrical interference. BIS should be interpreted cautiously in patients with neurological disorders and those taking psychoactive medications.

7) With **deepening anesthesia**, apart from a BIS decrease, there are **changes in **other **EEG-derived parameters **that are readily available on the monitor:

**a. EEG **may be set on a 50 μV scale at a sweep speed of 50 mm/sec. A typical adult EEG signal is 10 μV to 100 μV in amplitude when measured from the scalp. Simplistically, the EEG shows slowing (decreasing frequency in Hz) and increasing amplitude with deepening anesthesia.

**i. Gamma **(30-70 Hz)

**ii. Beta **(12-30 Hz)

**iii. Alpha **(8-12 Hz), sleep spindles, K complexes

**iv. Theta **(4-8 Hz)

**v. Delta **(< 4 Hz)

**vi. Burst suppressionvii. Iso-electricityb. SEF **- The frequency (from 0.5 - 30.00 Hz) at which 95% of the total EEG power lies below it and 5% lies above it. Lower SEFs are in keeping with deeper anesthesia.

**c. SR **- There may be periods of flat EEG trace (burst suppression), which may progress to a flat line or isoelectricity. Suppression ratio (SR) is the % of time (0-100%) over the last 63-sec period that the EEG signal is in the "suppressed" state.

8) When there is excessive muscle activity, there is a possibility that the BIS may be falsely elevated. The **EMG **shows the power (from 30-80 decibels) in the frequency range 70 - 110 Hz.

**9) SQI **is a calculated measure of the signal quality (from 0-100) for the EEG channel source(s).

**10) BIS Smoothing Rate **should be set at 15 sec. to provide increased responsiveness to state changes, such as induction or awakening.

### ETAG Protocol

1) The protocol is intended as a **rough guide**, not to be prescriptive.

2) ETAG **alarms **are set to alert, not to restrict.

3) On some machines, anesthesia gas alarms are available only for **inspired anesthetic gas**. With low flow anesthesia and when the anesthesia dose (vaporizer) is adjusted, there may be discrepancies between inspired anesthesia gas and end-tidal anesthesia gas (ETAG).

4) Ideally anesthesia should be titrated such that the ETAG is maintained between **0.7 and 1.3 **age-adjusted MAC during surgery. During **skin closure**, the anesthetic **gas may be turned **down at the discretion of the anesthesia practitioner.

**5) MAC-awake **is about 1/3 MAC for desflurane, sevoflurane and isoflurane.

6) There is evidence that at twice MAC-awake (about 0.7 MAC), even **distressing **(auditory) stimuli are not internalized.

7) The ETAG should be **interpreted in context**, taking into account patient characteristics, surgical stimulation, hemodynamic parameters, analgesic administration, and use of regional anesthesia.

**8) Age **has an important bearing on MAC.

9) The **age-adjusted MAC **values [and 0.7-1.3 MAC ranges] are given below:

(At sea level, 1 KPa anesthetic gas is approximately equal to 1%)

**a. Desflurane**:

i. Age 18-39: 7 KPa [4.9 to 9.1 KPa]

ii. Age 40-59: 6 KPa [4.2 to 7.8 KPa]

iii. Age 60-79: 5.2 KPa [3.6 to 6.8 KPa]

iv. Age ≥80: 4.55 KPa [3.2 to 5.9 KPa]

**b. Sevoflurane**:

i. Age 18-39: 2.4 KPa [1.7 to 3.1 KPa]

ii. Age 40-59: 1.7 KPa [1.2 to 2.2 KPa]

iii. Age 60-79: 1.55 KPa [1.1 to 2.0 KPa]

iv. Age ≥80: 1.2 KPa [0.8 to 1.6 KPa]

**c. Isoflurane**:

i. Age 18-39: 1.3 KPa [0.9 to 1.7 KPa]

ii. Age 40-59: 1.1 KPa [0.8 to 1.4 KPa]

iii. Age 60-79: 1.0 KPa [0.7 to 1.3 KPa]

iv. Age ≥80: 0.8 KPa [0.6 to 1.0 KPa]

10) You should be blinded to the BIS number, the EEG trace, and all the EEG-derived parameters, apart from the **SQI **(signal quality index).

## Appendix 2: Postoperative Questionnaire

Title of Study: BAG-RECALL: BIS or Anesthesia Gas to Reduce Explicit RecallExtubation date: ____________________________ Interviewer's Initials________

Today's date: ____________________________________

1. **What is the last thing you remember before going to sleep?**

-Being in the pre-op area

-Seeing the operating room

-Being with family

-Hearing voices

-Feeling mask on face

-Smell of gas

-Burning or stinging in the IV line

-Other [Free Text]: _______________________________________________

2. **What is the first thing you remember after waking up?**

-Hearing voices

-Feeling breathing tube

-Feeling mask on face

-Feeling pain

-Seeing the operating room

-Being in the recovery room

-Being with family

-Being in ICU

-Nothing

-Other [Free Text]: _______________________________________________

3. **Do you remember anything between going to sleep and waking up?**

-No

**-Yes**:-Hearing voices

-Hearing events of the surgery

-Unable to move or breathe

-Anxiety/stress

-Feeling pain

-Sensation of breathing tube

-Feeling surgery without pain

-Other [Free Text]: ______________________________________________

4.) Did you dream during your procedure?

-No

-Yes

-What about [Free Text]: _______________________________

5.) Were your dreams disturbing to you?

-No

-Yes

6.) Did you experience any nausea or vomiting following your operation?

-No

-Yes

If yes, how many times? _________

7.) What was the worst thing about your operation?

-Anxiety

-Pain

-Recovery process

-Functional limitations

-Awareness

-Other [Free Text]: _______________________________________________

8.) Are you left or right handed?

-left

-right

9.) Are you a natural red head?

- Yes

- No

10.) Regarding your regular sleep, how often do you remember your dreams?

- Never

-Every few weeks

-At least once a week

-Daily

11.) Do you make any effort to remember your dreams?

-No

-Yes

If yes, how?________________________________________________________

## Appendix 3: Structured follow-up questionnaire for patients who report memories of the period between "going to sleep" and "waking up" at either routine postoperative interview

1) When we contacted you about your surgery, you mentioned that you remembered something between going to sleep at the beginning of your surgery and waking at the end of your surgery. Do you still have memories of events that occurred during your surgery?

2) What do you remember?

a. Did you hear anything? (What?)

i) Voices (gender)

ii) Specific words

iii) Music

iv) Other

b. Did you any emotions? (What?)

i) Happiness

ii) Fear

iii) Calm

iv) Helplessness

v) Other

c. Did you experience any sensations?

i) Warmth

ii) Pressure

iii) Cold

iv) Pain

v) Other

d. Did you try to move? If yes, could you?

e. What was your breathing like?

i) Normal

ii) Fast

iii) Labored

iv) Unable to breathe

v) Other

f. Did you see anything? What did you see?

i) Light

ii) Colors

iii) Shapes

iv) Specific image

v) Other

g. Did you try to open your eyes? If yes, could you?

h. Were you afraid?

i. Did the experience distress you?

3) Do these memories trouble you?

4) If yes, how do they trouble you?

a. Have you experienced stress?

b. Have you felt any negative emotions because of them?

i) Fear

ii) Helplessness

iii) Anger

iv) Frustration

v) Other

c. Do you avoid any situations as a result of your experiences?

d. Do you experience waking flashbacks?

e. Do you have bad dreams?

f. Has your social life been affected?

5) Did you go to the intensive care unit after your surgery? If yes, did you still have the breathing tube in?

6) Did your awareness experience occur before the start of the surgery, during the surgery or when you were waking up from the surgery?

7) Do you think your awareness experience took place in the operating room or in the intensive care unit or both? Why do you think this?

8) Have you spoken to a health counselor about these experiences?

9) Do you suffer from insomnia?

10) Are you easily awakened from regular sleep?

11) Do you remember your dreams during regular sleep?

12) Would you like to speak to a professional about your experiences? We can give you a number of a professional who can see you.

13) Is it all right with you if we contact you again to talk to you about your experiences?

## Pre-publication history

The pre-publication history for this paper can be accessed here:

http://www.biomedcentral.com/1471-2253/9/8/prepub
